# Complete Genome Sequence of Bifidobacterium adolescentis ZJ2, Isolated from a Centenarian in Anhui, China

**DOI:** 10.1128/MRA.00710-20

**Published:** 2020-07-16

**Authors:** Zeyu Jin, Wei Li, Wei Wang, Baolin Sun

**Affiliations:** a Chorain Health Technology Co., Ltd., Hefei, Anhui, China; b School of Life Science, University of Science and Technology of China, Hefei, Anhui, China; University of Rochester School of Medicine and Dentistry

## Abstract

In this announcement, we present the complete genome sequence of Bifidobacterium adolescentis strain ZJ2, which was isolated from a female centenarian in Anhui, China. The final genome consists of a 2,401,766-bp chromosome with a G+C content of 59.90%.

## ANNOUNCEMENT

*Bifidobacterium* is the most common genus found in the gastrointestinal tract in both infants and adults (1). The abundances of *Bifidobacterium* species in human intestine vary with aging, and Bifidobacterium adolescentis is one of the predominant *Bifidobacterium* groups in the adult gut microbiota (2). It has been reported that B. adolescentis possesses extensive capabilities to affect the metabolism of cholesterol, to improve intestinal digestion, and to enhance immune barrier functions in mouse models (3).

We isolated one Bifidobacterium longum strain from a centenarian previously (4); this time, we isolated B. adolescentis strain ZJ2 from a fecal sample from a 105-year-old female subject from Bozhou, Anhui, China. Written informed consent was obtained, and no further ethics approval was needed according to local regulations. The fecal sample was serially diluted with normal saline (0.9% NaCl), and 0.1-ml aliquots of the appropriate dilutions were plated onto commercial MRS agar medium (Oxoid, Basingstoke, UK) supplemented with 0.5 g/liter l-cysteine or onto TPY agar medium (Hopebio, Qingdao, China) under anaerobic conditions (10% [vol/vol] H_2_, 5% [vol/vol] CO_2_, and 85% [vol/vol] N_2_) at 37°C for 24 to 48 h. The milky-white round colonies on both plates were picked and identified with the Vitek 2 ANC card. The inoculation suspension was prepared with 0.45% sodium chloride, and the turbidities were up to 2.70 to 3.30 McFarland standards using a calibrated Vitek 2 DensiCHEK instrument (bioMérieux, Marcy l’Etoile, France). The colonies were anaerobically incubated in TPY broth medium (Hopebio) at 37°C for 16 to 18 h. Genomic DNA was extracted by using a StarSpin bacterial DNA kit (GenStar, Beijing, China). PCR was performed using the universal primers 27F (5′-AGA GTT TGA TCC TGG CTC AG-3′) and 1492R (5′-TAC GGC TAC CTT GTA CGA CTT-3′). In order to identify the phylogenetic positions of these bacterial strains, the full lengths of the 16S rRNA genes were sequenced. The resulting sequences were automatically aligned, inspected by eye, and compared using BLAST (http://blast.ncbi.nlm.nih.gov/Blast.cgi).

The complete genome sequencing of B. adolescentis strain ZJ2 was performed using a PacBio Sequel II platform and an Illumina HiSeq 4000 platform (TruSeq DNA PCR-free 350-bp library) at the Beijing Genomics Institute (Shenzhen, China). For the Illumina sequencing library, the insert size was 350 bp, with a paired-end sequencing length of 150 bp; 1 μg genomic DNA was randomly fragmented using a g-TUBE device (Covaris, Inc.) following the manufacturer’s instructions. Fragments of 200 to 400 bp were selected using magnetic beads, and after end repair, 3ʹ adenylation, and adapter ligation, PCR products were purified with magnetic beads. Then, the double strands were heat denatured to construct the final library. For the PacBio sequencing library, the insert size was longer than 10 kb; 1 μg genomic DNA was sheared into 10- to 15-kb fragments using a g-TUBE device. The SMRTbell Express template preparation kit v2.0 (Pacific Biosciences, USA) was used for single-strand overhang removal, DNA damage repair, end repair, 3ʹ adenylation, and adapter ligation. AMPure PB Beads were used for fragment purification, and the BluePippin size selection system (Sage Science, USA) was used to select fragments longer than 10 kb. The Qubit DNA high-sensitivity (HS) assay kit in a Qubit fluorometer (Thermo Fisher Scientific, MA) was used for library quantification, and the Agilent 2100 bioanalyzer system with an Agilent HS DNA kit (Agilent Technologies, CA) was used to check the size of the library. The library was then sequenced using the Sequel sequencing kit v2.0 (PacBio).

In total, 9,797,196 Illumina paired-end raw reads were generated and corrected (filtered bases with a quality score lower than 20, >40%; N content, >10%). In addition, 84,026 PacBio subreads were generated and corrected (subreads with less than 1 kb were removed). The *N*_50_ value for the PacBio subreads is 10,104 bp, and the *N*_90_ value is 4,529 bp. Self-correction was performed using proovread v2.12 (https://github.com/BioInf-Wuerzburg/proovread) (t, 4; coverage, 60; mode, sr). Celera Assembler v8.3 (5) (doTrim_initialQualityBased, 1; doTrim_finalEvidenceBased, 1; doRemoveSpurReads, 1; doRemoveChimericReads, 1; d properties, U) with other default settings was used for draft unitig assembly, overlap identification and trimming, and closed circular genome construction. Single-base corrections were then performed using GATK v1.6-13 (6) (cluster, 2; window, 5; stand_call_conf, 50; stand_emit_conf, 10.0; dcov, 200; MQ0, ≥4).

Based on the assembly information, the ZJ2 complete genome consists of 2,401,766 bp, with a G+C content of 59.90%. The genomes were annotated with Glimmer3 v3.02 (7) (o, *; g, *; t, *; l, linear), tRNAscan-SE v1.3.1 (8) [Spec_tag(BAOG); o, *.tRNA; f, *.tRNA.structure], RNAmmer v1.2 (9) (s, species; m, type; gff, *.rRNA.gff; f, *.rRNA.fq), and the Rfam database v9.1 (10) (p, blastn; W, 7; e, 1; v, 10,000; b, 10,000; m, 8; i, subfile; o, *.blast.m8). A total of 2,083 putative protein-coding genes were predicted, along with 13 rRNA genes, 0 tRNA genes, and 2 small RNA genes. MUMmer v3.22 (11) (b, 200; c, 65; extend; l, 20) was used for synteny analysis of B. adolescentis strain ZJ2 and four other B. adolescentis strains (22L, ATCC 15703, BBMN23, and P2P3); 1,227 core genes of these five strains were identified using CD-HIT v4.6.6 (12) (c, 0.5; n, 3; p, 1; g, 1; d, 0; s, 0.7; aL, 0.7; aS, 0.7).

### Data availability.

This whole-genome project has been deposited in DDBJ/EMBL/GenBank under accession number CP047129.1. The BioProject accession number is PRJNA565810. Raw sequence reads have been deposited in the SRA under accession numbers SRX8107993 and SRX8108009.
